# The predictive role of serum calprotectin on mortality in hemodialysis patients with high phosphoremia

**DOI:** 10.1186/s12882-020-01812-x

**Published:** 2020-05-04

**Authors:** Tomoko Kanki, Takashige Kuwabara, Jun Morinaga, Hirotaka Fukami, Shuro Umemoto, Daisuke Fujimoto, Teruhiko Mizumoto, Manabu Hayata, Yutaka Kakizoe, Yuichiro Izumi, Saeko Tajiri, Tetsuya Tajiri, Kenichiro Kitamura, Masashi Mukoyama

**Affiliations:** 1 Department of Nephrology, Graduate School of Medical Sciences, Kumamoto University, 1-1-1 Honjo, Chuo-ku, Kumamoto, Japan; 2 Department of Clinical Investigation, Kumamoto University Hospital, 1-1-1 Honjo, Chuo-ku, Kumamoto, Japan; 3 Medical Corporation, Jinseikai, 2-3-10 Toshima-nishi, Higashi-ku, Kumamoto, Japan; 4 Third Department of Internal Medicine, Faculty of Medicine, University of Yamanashi, 1110 Shimokato, Chuo, Yamanashi, Japan

**Keywords:** Serum calprotectin, Chronic inflammation, Prognostic biomarker, Hemodialysis patients, Phosphate

## Abstract

**Background:**

The inflammatory mediator calprotectin (CPT, myeloid-related protein 8/14) is known as an endogenous ligand contributing to pathophysiology in inflammatory diseases. Serum CPT reportedly became a potential biomarker in these conditions, though there is no report predicting the prognosis in hemodialysis patients. The aim of this study is to investigate the predictive role of serum CPT on mortality in hemodialysis patients.

**Methods:**

We conducted a multicenter, observational cohort study of 388 Japanese subjects undergoing hemodialysis. Serum CPT were measured using an ELISA. The potential associations between serum CPT and clinical variables were cross-sectionally examined. Multivariate Cox regression was used to estimate the association between serum CPT, high-sensitivity C reactive protein (hs-CRP), white blood cell (WBC) count and mortality. Median follow-up was 6.6 years.

**Results:**

The median CPT level was 6108 ng/ml (median in healthy subjects, 2800) at baseline. Serum CPT positively correlated with WBC count (ρ = 0.54, *P* < 0.001) and hs-CRP values (ρ = 0.35, P < 0.001). In multivariate analysis, hs-CRP was an independent predictor of all-cause mortality after adjusting confounding factors (middle vs. low: hazard ratio [HR] 2.09, 95% confidence interval [CI] 1.23–3.66; high vs. low: 2.47, 1.40–4.47). In the analysis by stratum of phosphate levels, elevated CPT levels were significantly associated with all-cause mortality in the highest tertile (18.1; 3.15–345.9) among the high-phosphate group, but not among the low-phosphate group.

**Conclusions:**

Serum CPT would become a potential predictive marker on mortality in hemodialysis patients with high-phosphate levels.

## Background

Chronic inflammation has so far been known as an important comorbid condition contributing to the prognosis in hemodialysis (HD) patients [1, 2]. Such systemic and chronic inflammation in patients with end-stage renal disease (ESRD) could result in a malnutrition, inflammation and atherosclerosis (MIA) syndrome [3, 4]. In non-dialyzed patients with diabetes, obesity, cancer or autoimmune disorders, chronic inflammation is caused by interactions between pattern recognition receptors such as toll-like receptors and their endogenous ligands, collectively called damage-associated molecular patterns (DAMPs) [5]. In HD patients, low-grade endotoxemia could become an important factor to induce chronic inflammation in addition to DAMPs.

We and others recently revealed the pathogenic role of toll-like receptor 4 (TLR4) in the development and progression of diabetic nephropathy [6–8] and crescentic glomerulonephritis [9, 10]. With regard to DAMPs, myeloid-related protein 8 (MRP8, also known as S100A8 or calgranulin A) was originally identified as a cytoplasmic calcium-binding protein in neutrophils and monocytes [11]. MRP8 usually forms a heterodimeric complex myeloid-related protein 8/14 (MRP8/14), also named as calprotectin (CPT), with a binding partner myeloid-related protein 14 (MRP14, also known as S100A9 or calgranulin B) in the bloodstream [12]. Several key reports have shown that the CPT could act as a potent endogenous ligand for TLR4 in various diseases including septic shock, vascular injury, and autoimmune disorders [13–15]. In human pathologies, CPT was reportedly involved in several renal diseases including diabetic nephropathy, crescentic glomerulonephritis and membranoproliferative glomerulonephritis [16–18]. Besides, as a biomarker, urinary CPT might become useful indicator to diagnose an acute kidney injury [19, 20]. In Chinese patients with peritoneal dialysis, serum CPT level was associated with lower survival and a trend of less cardiovascular event-free period [21]. To our knowledge, however, there is no cohort study investigating the prognostic role of serum CPT in HD patients.

In this cohort, we evaluated the predictive role of serum CPT levels on all-cause mortality in HD patients.

## Methods

### Study design and subjects

This study was conducted with an observational, multicenter retrospective cohort design using the data previously performed prospective study in our lab [22]. Briefly, a total of 412 patients treated for ESRD at the Jinseikai clinic by regular dialysis treatment were enrolled in this study. All patients provided their written informed consent in 2012, before participating in the study. All subjects underwent 6 and a half year of prospective follow-up except for those who died, received kidney transplantation, or transferred to a dialysis unit outside the Jinseikai clinic during follow-up period. These collected data were retrospectively analyzed with serum CPT measurement in 2018. After exclusion of 12 subjects due to missing serum samples, we measured serum CPT levels in 400 subjects. Of those 400 subjects, 12 showed missing clinical information, including 6 subjects lacking pre-dialysis blood pressure, 2 subjects lacking cardiothoracic ratio, 1 subject lacking body mass index (BMI), 3 subjects lacking smoking habits, leaving 388 subjects (Figure S1). This study was conducted in keeping with Helsinki Declaration and with approval of ethics committees for clinical research at Kumamoto University (No.1255-R).

### Data collection

Demographic characteristics of the subjects, comorbid conditions, and laboratory data were obtained at study entry. The primary outcome was mortality and the clinical events were identified prospectively. The information regarding death was classified into cardiovascular, infection-related, or cancer death based on electronic medical records confirmed by physicians responsible for conducting the report. Cardiovascular mortality was defined as death from ischemic heart disease, cerebrovascular disease, congestive heart failure, arrhythmia, aortic disease, or sudden death.

### Clinical evaluation and laboratory testing

Serum samples that were obtained at the initiation of the first outpatient HD session in the week and that would otherwise have been discarded after routine clinical testing were saved and stored at − 80 °C in 2012.

Smoking habits were defined as smoking status at baseline. Obesity was defined as BMI ≥ 25 kg/m^2^. Hypertension was defined as past history, systolic blood pressure (SBP) ≥140 mmHg, or diastolic blood pressure (DBP) ≥90 mmHg at baseline. Diabetes was defined as past history of diabetes, or serum glucose ≥200 mg/dl. Dyslipidemia was defined as past history, or triglycerides ≥150 mg/dl, low density lipoprotein cholesterol (LDL-Cho) ≥140 mg/dl, or high-density lipoprotein cholesterol (HDL-Cho) < 40 mg/dl. Coronary artery disease, stroke, peripheral artery disease (PAD), rheumatoid arthritis (RA) were defined as past history of disease and active cancer was defined as onset of cancer within 5 years before the entry. Statin was defined as having been prescribed statin. RAS inhibitor was defined as prescription of an angiotensin-converting enzyme inhibitor or an angiotensin II receptor blocker. Cardiovascular disease (CVD) included acute myocardial infarction (MI), silent MI, or coronary artery disease followed by coronary artery bypass surgery or angioplasty. Silent MI was defined as myocardial scarring without any historical indication of clinical symptoms or abnormal cardiac enzyme changes [23]. All laboratory data except for CPT levels were gathered in 2012. In 2018, serum CPT protein levels were measured, using samples collected in 2012 and stored at − 80 °C, at the Department of Nephrology, Kumamoto University, using a human CPT sandwich enzyme-linked immune-sorbent assay (ELISA) kit [BioLegend, San Diego, CA, USA].

### Statistical analyses

Wilcoxon rank sum test or Spearman’s correlation coefficients were estimated to determine associations between serum CPT levels and clinical parameters for categorical or continuous data, respectively. With regard to baseline characteristics, continuous data with normal distribution were summarized as means (standard deviation [SD]), continuous variables with skewed data as medians (inter-quartile range [IQR]), and categorical data as proportions. We used the Kaplan–Meier method to estimate the crude probability of all-cause mortality associated with tertiles of CPT levels, and multivariate Cox regression to examine the adjusted relationship between CPT levels and mortality. In accordance with clinical implication and cross-sectional analysis, adjustment was made for expanding sets of covariates: age, sex, diabetes, rheumatoid arthritis (RA), active cancer, history of coronary artery disease, albumin, Cr and inflammatory parameters including CPT, hs-CRP and WBC. Continuous variables with skewed distribution were log-transformed to attain normal distribution. Statistical method for each result is summarized in supplemental S1 Table. All statistical analysis was performed using JMP Pro 12.0.0 software (SAS Institute, Cary, NC) and EZR (Saitama Medical Center, Jichi Medical University, Saitama, Japan), a graphical user interface for R 2.13.0 (R Foundation for Statistical Computing, Vienna, Austria). All *p*-values were two-tailed, and *P* < 0.05 was taken as statistically significant.

## Results

### Baseline characteristics and causes of death in this cohort

The baseline characteristics of the participants are summarized in Table 1. The mean age was 65.3 ± 12.6 years and the median duration of dialysis before study entry was 5.79 (2.93–12.12) years. The two major cause of ESRD in this cohort were diabetic nephropathy (35%) and glomerulonephritis (30%). Serum CPT level in our patients undergoing HD was 6108 (4017–8906) ng/ml, which showed higher than that of healthy subjects (median 2800 ng/ml, reported in other previous study [17]). No patients exhibited active inflammation (hs-CRP ≥ 1.0 mg/dl and WBC ≥ 10,000 /μl).

**Table 1:** Baseline characteristics of the cohort

	Total *n* = 388
Variable	n (%), mean (±SD) or median (IQR)
Age, years	65.3 (±12.6)
Female gender, n (%)	143 (36.9)
Follow-up duration, years	6.62 (3.76–6.64)
Vintage, years	5.79 (2.93–12.12)
Systolic BP, mmHg	146.7 (±20.3)
Diastolic BP, mmHg	77.3 (±13.8)
BMI, kg/m^2^	21.7 (±3.5)
CTR, %	49.0 (±5.3)
Single-pool Kt/V	1.44 (±0.27)
ABI	1.10 (0.99–1.17)
Smoking habits, n (%)	64 (16.5)
RAS inhibitor, n (%)	207 (53.3)
Statin, n (%)	79 (20.4)
Chronic kidney disease etiology, n (%)	
Diabetic nephropathy	136 (35.1)
Hypertensive disease	31 (8.0)
Glomerulonephritis	118 (30.4)
Polycystic kidney disease	10 (2.6)
Others	93 (24.0)
Comorbid conditions, n (%)	
Coronary artery disease (CAD)	56 (14.4)
Stroke	64 (16.5)
Peripheral Arterial Disease	81 (20.9)
Active cancer	20 (5.2)
Rheumatoid arthritis (RA)	5 (1.3)
Diabetes mellitus (DM)	159 (41.0)
Hypertension	319 (82.2)
Dyslipidemia	198 (51.0)
Laboratory findings	
WBC, /μl	5626 (±1666)
hs-CRP, mg/l	0.708 (0.295–1.828)
Hemoglobin, g/dl	10.7 (±1.1)
Plt, 10^4^/μl	16.4 (±4.7)
Albumin, g/dl	3.8 (±0.33)
BUN, mg/dl	61.0 (±14.7)
Cr, mg/dl	10.9 (±2.6)
Whole-PTH, pg/ml	45 (24–92)
aCa, mg/dl	9.4 (±0.72)
IP, mg/dl	5.5 (±1.2)
LDL-cholesterol, mg/dl	79.3 (±23.6)
TG, mg/dl	84 (59–126)
CPT, ng/ml	6108 (4017–8906)

*SD* standard deviation, *IQR* inter quartile range, *BP* blood pressure, *BMI* body mass index, *CTR* cardio-thoracic ratio, *KT/V* dialysis dose, *ABI* Ankle-Brachial-Index, *Hb* hemoglobin, *Plt* platelets, *BUN* blood urea nitrogen, *Cr* Creatinine, *Whole-PTH* whole parathyroid hormone, *aCa* adjusted calcium, *IP* inorganic phosphorus, *LDL-Cho* low density lipoprotein cholesterol, *TG* triglyceride, *CPT* calprotectin

During the median follow-up of 6.6 years, 118 (30.4%) deaths occurred. Infectious disease (33.9%) was the most common cause of death, followed by cardiovascular disease (22.9%) (S2 Table).

### Cross-sectional analysis at baseline

**Fig. 1:**
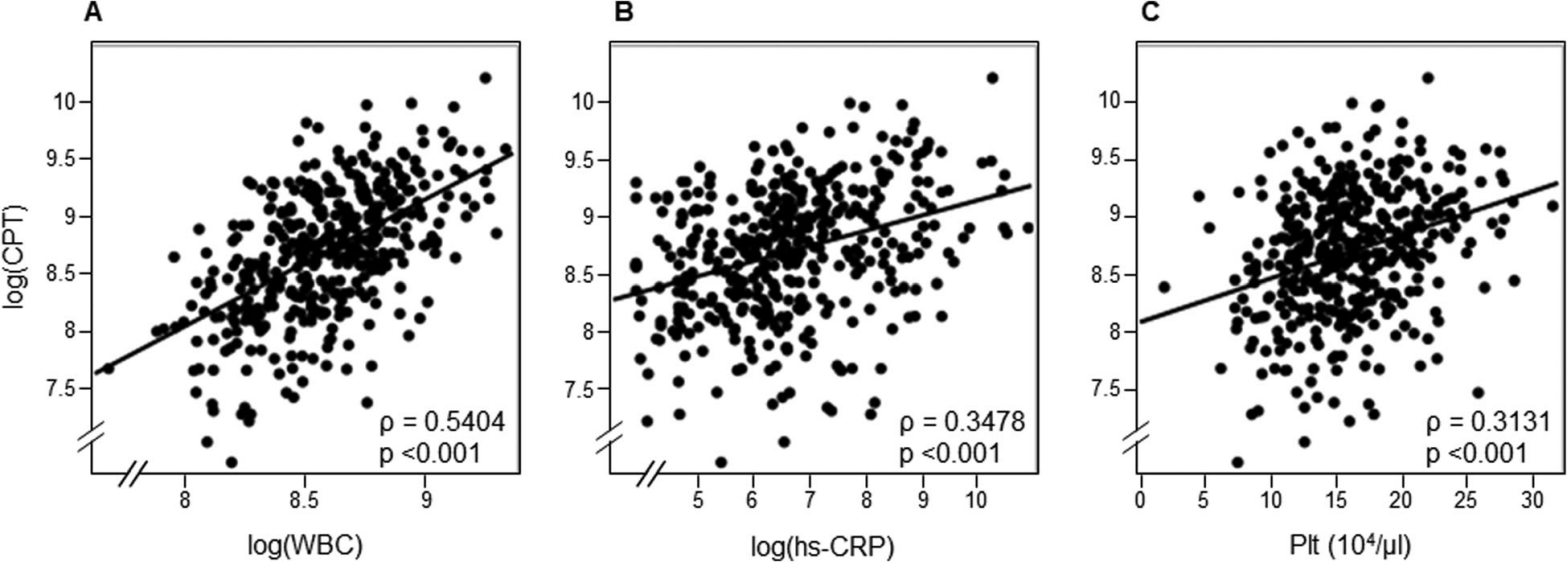
2D-scatter plot between clinical parameters and CPT at baseline. Correlation between CPT and WBC (**a**), hs-CRP (**b**) and Plt (**c**). CPT, calprotectin; WBC, white blood cells; hs-CRP, high sensitivity C-reactive protein; Plt, platelets

In Tables 2 and 3, the association between serum CPT and the other clinical parameters were cross-sectionally analyzed at baseline. In categorical variables, serum CPT was significantly higher in male or subjects with RA (Table 2). In continuous parameters, serum CPT was positively correlated with white blood cells (ρ = 0.54, *P* < 0.001), hs-CRP values (ρ = 0.35, *P* < 0.001) and also weakly with platelet count (ρ = 0.31, *P* < 0.001) (Table 3). The distributions between serum CPT and these inflammatory parameters were indicated in Fig. 1. There were also significant association with BMI, serum inorganic phosphorus and triglyceride (*P* < 0.001), but all of these correlation coefficients were below 0.3, suggesting not very meaningful (Tables 3 and Figure S2).

**Table 2:** Association between categorical variables and CPT level at baseline

categorical variables		n	median CPT (IQR)	*p*-values
Gender	male	245	6316 (4289–9501)	0.0100
	female	143	5631 (3539–8132)	
Active cancer	–	368	6193 (4080–8913)	0.2658
	+	20	5134 (3009–8845)	
Rheumatoid arthritis (RA)	–	383	6052 (3996–8818)	0.0169
	+	5	10,241 (7948–15,360)	
Diabetes mellitus (DM)	–	229	5807 (3851–9379)	0.2955
	+	159	6392 (4318–8643)	
History of coronary artery disease	–	332	6058 (4080–8819)	0.6530
	+	56	6654 (3866–9403)	
Smoking habits	–	324	6287 (3996–8917)	0.8567
	+	64	5879 (4288–8701)	
Stroke	–	324	6108 (4017–8819)	0.8486
	+	64	6130 (4019–9212)	
Peripheral Arterial Disease	–	307	6173 (3970–8920)	0.6954
	+	81	6064 (4186–8836)	

*CPT* calprotectin, *IQR* inter quartile range

**Table 3:** Association between continuous parameters and CPT level at baseline

continuous variables	Spearman‘s correlation coefficient (ρ)	*p*-values
Age (years)	−0.0815	0.109
Vintage (years)	−0.1684	< 0.001
BMI (kg/m^2^)	0.1846	< 0.001
systolic BP (mmHg)	−0.0145	0.7761
diastolic BP (mmHg)	0.0796	0.1173
CTR (%)	− 0.1715	< 0.001
Kt/V	−0.1622	0.0013
ABI	−0.0594	0.2453
WBC (/μl)	0.5404	< 0.001
hs-CRP (mg/l)	0.3478	< 0.001
Hb (g/dl)	0.1433	0.0047
Plt (10^4^/μl)	0.3131	< 0.001
Albumin (g/dl)	0.0889	0.0802
Cr (mg/dl)	0.1340	0.0082
Whole-PTH (pg/ml)	0.0332	0.5148
aCa (mg/dl)	−0.0506	0.3200
IP (mg/dl)	0.2165	< 0.001
LDL-cholesterol (mg/dl)	−0.0042	0.9340
TG (mg/dl)	0.2599	< 0.001

*CPT* calprotectin, *BMI* body mass index, *BP* blood pressure, *CTR* cardio-thoracic ratio, *KT/V* dialysis dose, *ABI* Ankle-Brachial-Index, *WBC* white blood cell, *hs-CRP* high sensitivity C-reactive protein, *Hb* hemoglobin, *Plt* platelets, *Cr* Creatinine, *Whole-PTH* whole parathyroid hormone, *aCa* adjusted calcium, *IP* inorganic phosphorus, *LDL-Cho* low density lipoprotein cholesterol, *TG* triglyceride

### Association of individual inflammatory markers with all-cause mortality: Kaplan–Meier method and cox proportional hazard models

**Fig. 2:**
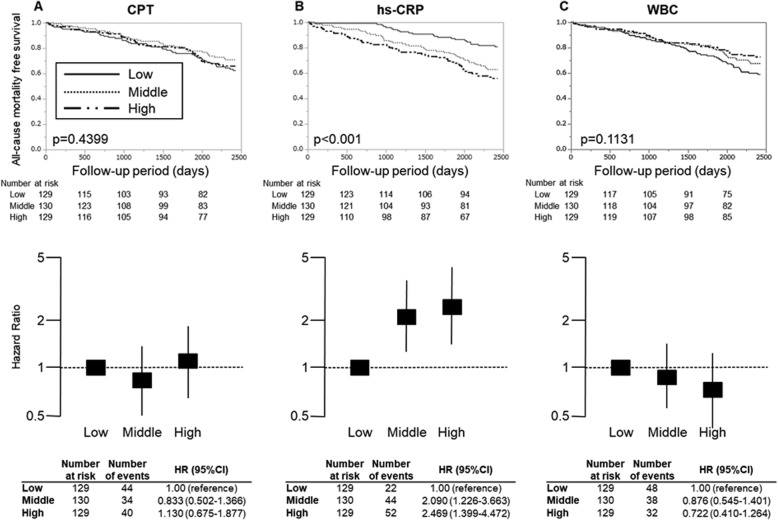
Survival curve and mortality risk according to CPT, hs-CRP and WBC tertiles for total cohort. Kaplan–Meier survival probability and multivariate adjusted hazard ratios in all-cause mortality are calculated according to CPT, hs-CRP and WBC tertiles for the total cohort. p-values report the test for trend. **a** CPT; **b** hs-CRP; **c** WBC. In multivariate analysis, the model was adjusted for relevant covariates: age, sex, diabetes, rheumatoid arthritis, active cancer, history of coronary artery disease, albumin, Cr and inflammatory parameters. CPT, calprotectin; hs-CRP, high sensitivity C-reactive protein; WBC, white blood cells; Cr, Creatinine; HR, hazard ratio; 95% CI, 95% confidence interval. Tertiles of CPT, < 4635.04, 4635.04–7915.68, > 7915.68 ng/ml; hs-CRP, < 0.427, 0.427–1.173 and > 1.173 mg/l; WBC, < 4756.67, 4756.67–6156.67, > 6156.67 /μl

**Fig. 3:**
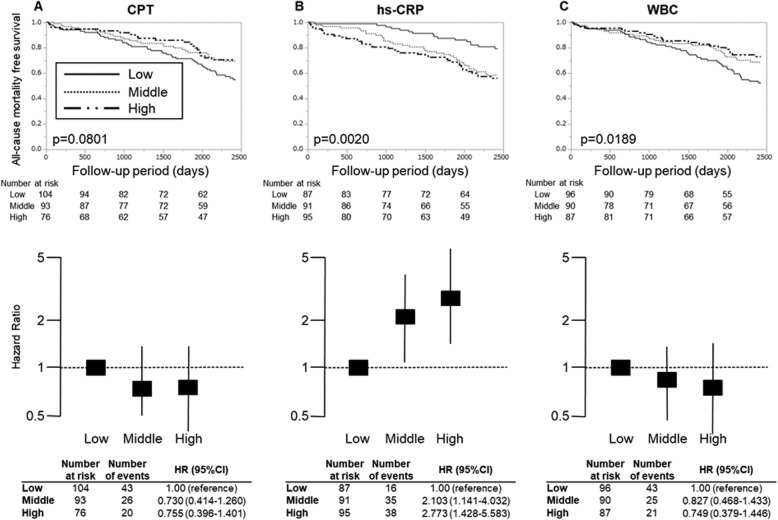
Survival curve and mortality risk according to CPT, hs-CRP and WBC tertiles in stratum of IP < 6.0 mg/dl. Kaplan–Meier survival probability and multivariate adjusted hazard ratios in all-cause mortality are calculated according to CPT, hs-CRP and WBC tertiles for the stratum of IP < 6.0 mg/dl. *p*-values report the test for trend. **a** CPT; **b** hs-CRP; **c** WBC. In multivariate analysis, the model was adjusted for relevant covariates: age, sex, diabetes, rheumatoid arthritis, active cancer, history of coronary artery disease, albumin, Cr and inflammatory parameters. CPT, calprotectin; hs-CRP, high sensitivity C-reactive protein; WBC, white blood cells; IP, inorganic phosphorus; Cr, Creatinine; HR, hazard ratio; 95% CI, 95% confidence interval. Tertiles of CPT, < 4635.04, 4635.04–7915.68, > 7915.68 ng/ml; hs-CRP, < 0.427, 0.427–1.173, > 1.173 mg/l; WBC, < 4756.67, 4756.67–6156.67, > 6156.67 /μl

**Fig. 4:**
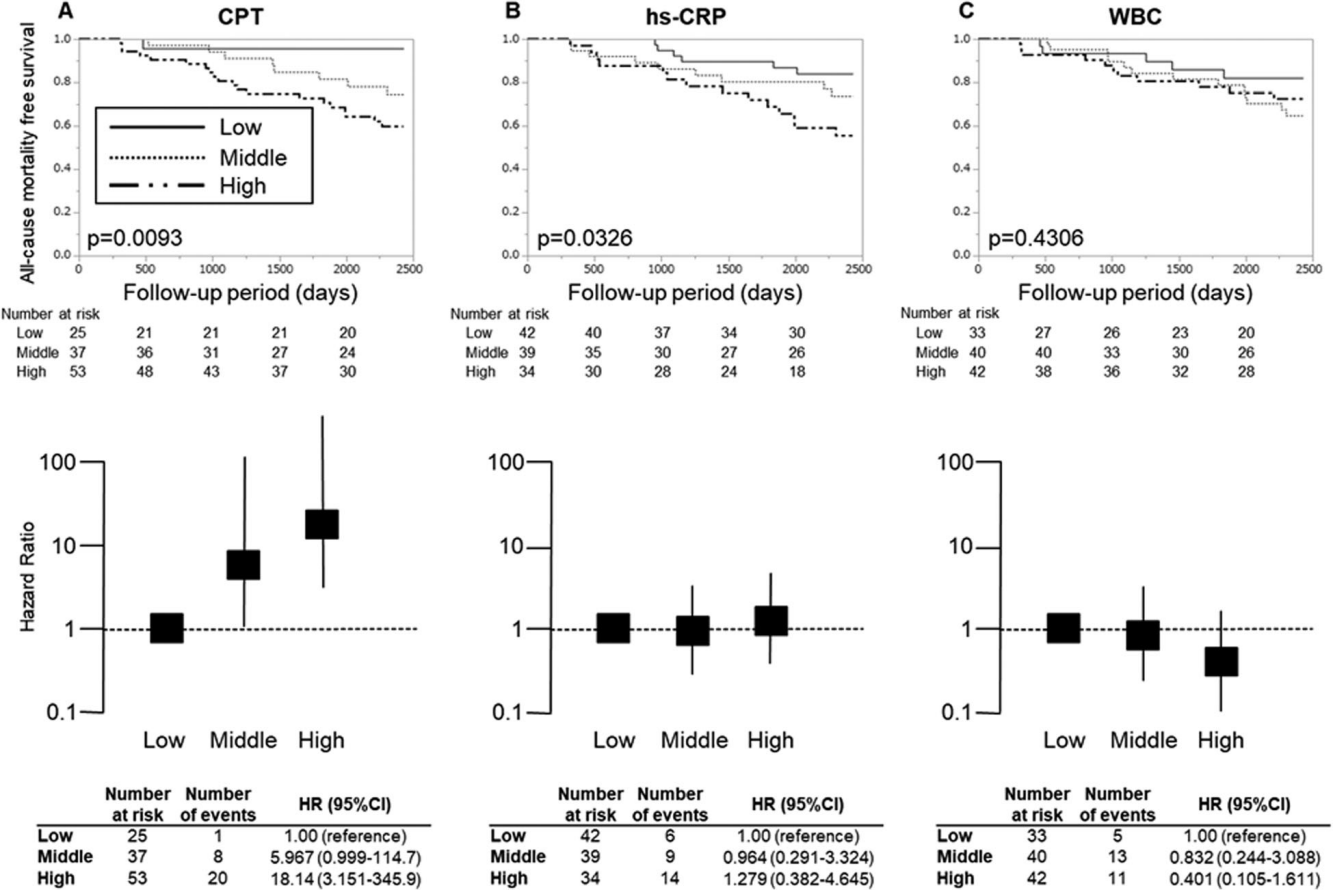
Survival curve and mortality risk according to CPT, hs-CRP and WBC tertiles in stratum of IP ≥ 6.0 mg/dl. Kaplan–Meier survival probability and multivariate adjusted hazard ratios in all-cause mortality are calculated according to CPT, hs-CRP and WBC tertiles for the stratum of IP ≥ 6.0 mg/dl. p-values report the test for trend. **a** CPT; **b** hs-CRP; **c** WBC. In multivariate analysis, the model was adjusted for relevant covariates: age, sex, diabetes, rheumatoid arthritis, active cancer, history of coronary artery disease, albumin, Cr and inflammatory parameters. CPT, calprotectin; hs-CRP, high sensitivity C-reactive protein; WBC, white blood cells; IP, inorganic phosphorus; Cr, Creatinine; HR, hazard ratio; 95% CI, 95% confidence interval. Tertiles of CPT, < 4635.04, 4635.04–7915.68, > 7915.68 ng/ml; hs-CRP, < 0.427, 0.427–1.173, > 1.173 mg/l; WBC, < 4756.67, 4756.67–6156.67, > 6156.67 /μl

We classified each inflammatory parameter including hs-CRP, WBC and CPT into tertile and performed Kaplan-Meier survival analysis (tertiles of CPT, < 4635.04, 4635.04–7915.68, > 7915.68 ng/ml; hs-CRP, < 0.427, 0.427–1.173, > 1.173 mg/l; WBC, < 4756.67, 4756.67–6156.67, > 6156.67 /μl). The hazard ratios (HRs) for all-cause mortality in comparison with each group of the lowest tertile were estimated by multivariate Cox proportional hazard models with adjusting confounding factors. In Fig. 2b, hs-CRP was an independent predictor of all-cause mortality after adjusting confounding factors (middle vs. low: HR 2.090, 95% confidence interval [CI] 1.226–3.663; high vs. low: HR 2.469, 95%CI 1.399–4.472). There was no significant difference in the tertiles of CPT and WBC (Fig. 2a and c). Because serum CPT levels reportedly depends on bone-marrow function [11] or nutritional status [24], we examined the possible modifications by such comorbid conditions upon HRs in all-cause mortality according to CPT, hs-CRP and WBC tertiles (Supplemental S3 Table). These analyses revealed that increasing serum CPT levels resulted in increasing mortality in the stratum of serum phosphate level > 6.0 mg/dl (Fig. 4a) rather than in total cohort (Fig. 2a). Stratification by serum phosphate level employed the cut-off value in accordance with guidelines edited by Japanese Society of Dialysis Therapy [25]. Kaplan–Meier survival curve and the HRs stratified by serum phosphate levels are shown in Figs. 3 and 4. In the stratum with low-phosphate levels, similarly to the crude analyses, none of the CPT level groups had any significant association with all-cause mortality (Fig. 3a). In contrast, in the stratum with high-phosphate levels, high-CPT groups were significantly associated with all-cause mortality by multivariate analysis [high vs. low: HR 18.14; 95%CI 3.151–345.9; significant interaction between serum phosphate levels and CPT levels (*P* = 0.029 based on the likelihood ratio test)] (Fig. 4a). Note, in the analysis of serum CPT as a continuous parameter, the increase of log-transformed serum CPT by 1 resulted in significant risk increase of prognosis (HR 8.406; 95%CI 2.383–29.65). This predictive significance was also confirmed by unadjusted- or age, sex adjusted-analyses in high-phosphate stratum. In the same analyses for hs-CRP and WBC, HRs did not become evident in the stratum with high-phosphate levels after adjustment for relevant covariates (Fig. 4b and c).

## Discussion

We investigated the predictive role of serum CPT levels on all-cause mortality in HD patients. Our multicenter cohort revealed that serum CPT was positively correlated with the inflammatory parameters also in HD patients as previous reports in non-dialysis patients with several inflammatory diseases, such as rheumatoid arthritis [26, 27], inflammatory bowel disease [28, 29], psoriasis [30] or chronic bronchitis [31]. It is noteworthy that the mortality or survival rate in HD patients with high-phosphate levels was well predicted by serum CPT levels. To our knowledge, this is the first report describing the role of serum CPT to predict prognosis in HD patients.

Chronic inflammation is one of the pivotal conditions contributing to the pathogenesis in HD patients. There are numerous factors inducing systemic chronic inflammation in HD patients, such as oxidative stress [32], uremic toxin, carbamylation for protein [33] or leukocyte activation by some dialyzers [34]. Highly-sensitive biomarker for inflammation, hs-CRP, could predict all-cause and cardiovascular death in HD patients [35] as well as in non-HD patients [36]. In our cohort, hs-CRP has also possessed the independent predictive power for all-cause mortality by multivariate Cox regression analysis (Fig. 2b). CPT is also inflammatory protein positively correlated to hs-CRP, WBC or platelet counts (Table 3 and Fig. 1). However, in crude analysis, serum CPT did not predict all-cause mortality rather than hs-CRP (Fig. 2a). In this study, stratified analyses disclosed the unknown aspects of serum CPT on mortality in the high-phosphate condition (Fig. 4a).

There is no direct evidence describing the association of CPT with high phosphate, but with inflammation. Extracellular phosphate exerts its cytotoxicity if it forms insoluble nanoparticles with calcium and fetuin-A, named as calciprotein particles (CPPs) [37]. In patients with chronic kidney disease (CKD), serum CPP levels are associated with serum phosphate concentration [38] and also with CKD stages [39]. CPP can lead to cellular damage through TLR4-dependent inflammatory cytokine secretion [40, 41]. We and others have reported that CPT exert its pathophysiological role due to amplifying the TLR4/nuclear factor-kappa B (NF-κB) signaling [13–16, 42]. Furthermore, CPT induced endothelial injury and atherothrombosis via platelet activation [43]. Hence, we hypothesize the direct and indirect mechanisms of CPT-related risk in the high-phosphate condition. As a direct effect, CPT possibly promotes the TLR4-dependent cytotoxicity induced by CPP. Perhaps, vascular lesion by FGF23-Klotho imbalance might be indirectly exacerbated in association with CPT-induced endotheliosis or platelet activation. Based on these hypotheses, we have also examined the role of Ankle-Brachial-Index (ABI) in this study. However, no significant results of ABI were observed in the cross-sectional analyses (Figure S3). As previously suggested, it should be noted that ABI is easily affected by its measurement condition such as pre−/post-dialysis or body-weight gain [44]. Otherwise, the calcification score evaluated by CT scan might be useful [25]. To elucidate the detailed roles of CPT affecting pathophysiology in the high-phosphate condition, the additional studies with the calcification score or CPP measurement will be warranted.

Seeing from another perspective, the low CPT level is obviously associated with a good survival rate in a group with sufficient nutritional status indicated by high BMI (S3 Table) as well as high phosphate levels (Fig. 4a). In this regard, low serum CPT could be a good predictor for survival in the high-phosphate group compared to hs-CRP or WBC (Fig. 4). Besides, the interaction term (phosphate and CPT) using the likelihood ratio test with Cox regression was significant (*P* = 0.029), suggesting that the stratification by phosphate level is meaningful. These results suggested that HD patients with well-nourished status might become “real” healthy if they have low CPT levels.

In a previous study, another inflammatory parameter, IL-6, was considered as a stronger predictor of total and cardiovascular mortality when compared to hs-CRP in HD patients [45]. Since IL-6 production is induced by CPT via TLR4 signaling that involves MAPK and NF-κB [46], analyzing the association between CPT and IL-6 is warranted to reveal a further role of CPT in mortality.

There are several limitations in our cohort. We could not determine the cause of death associated with high CPT, because the number of death endpoint was small. Besides, predictive role of CPT was analyzed by the measurement at single point of entry. In previous report, HD vintage was positively correlated with high serum CPT level [47], which should be considered as a crucial factor affecting the interpretation of prognostic biomarkers in HD patients. However, our results did not reproduce such correlation, partly due to the difference of population between studies. Subjects with acute inflammation were included in their previous study, but excluded in our study. Besides, our study showed that serum CPT predicted mortality in high-phosphate group but not in total cohort. It appears to be different from the other previous study of PD patients [21] for several reasons. First, since CRP level was higher than that in our cohort (3.43 vs. 0.708 mg/l), subjects affected by clinical and/or subclinical inflammation such as catheter-related exit-site or tunnel infection was possibly included. Second, dialysis method was different (peritoneal dialysis vs. hemodialysis). Third, the younger subjects were recruited in PD study (mean age in PD study and our study, 57.3 vs 65.3 years). Even though there was no data about serum phosphate levels in PD study, their younger population would match our high-phosphate group which mean age was more than 4 years younger than that in low-phosphate group (62.3 ± 13.6 vs 66.5 ± 11.9 years). Note, the information of phosphate binders and/or calcimimetics are missing in this study. We need the further analysis employing more population with multiple measurement during the follow-up period in future prospective cohort.

## Conclusions

Serum CPT would become a potential prognostic marker in HD patients with high-phosphate levels. This characteristic differs from that of a conventional inflammatory marker, hs-CRP.

## Supplementary information

**Additional file 1: S1 Table.** Statistical analyses. **S2 Table.** Causes of death in this cohort. **S3 Table.** Multivariate adjusted hazard ratios in all-cause mortality according to CPT, hs-CRP and WBC tertiles in strata by clinical parameters reflecting bone-marrow function and nutritional status. **Figure S1.** Flow chart of participants in this study. **Figure S2.** 2D-scatter plot between clinical parameters and CPT at baseline. **Figure S3.** 2D-scatter plot between ABI and CPT at baseline for total cohort and in stratum of IP.

## Data Availability

The datasets generated and/or analyzed during the current study are not publicly available, because the ethics committee for Clinical Research at Kumamoto University has placed restrictions on public data sharing because data contain sensitive information. However, data are available from the corresponding author on reasonable request.

## Abbreviations

HD: Hemodialysis
ESRD: End-stage renal disease
DAMPs: Damage-associated molecular patterns
TLR4: Toll-like receptor 4
MRP8: Myeloid-related protein 8
CPT: Calprotectin
MRP14: Myeloid-related protein 14
hs-CRP: High-sensitivity C reactive protein
BMI: Body mass index
CVD: Cardiovascular disease
ELISA: Enzyme-linked immune-sorbent assay
SD: Standard deviation
IQR: Inter-quartile range
RA: Rheumatoid arthritis
HR: Hazard ratio
CI: Confidence interval
CPPs: Calciprotein particles
NF-κB: Nuclear factor-kappa B
ABI: Ankle-Brachial-Index

## References

[1] Iseki K, Tozawa M, Yoshi S, Fukiyama K (1999). Serum C-reactive protein (CRP) and risk of death in chronic dialysis patients. Nephrol Dial Transplant.

[2] Zimmermann J, Herrlinger S, Pruy A, Metzger T, Wanner C (1999). Inflammation enhances cardiovascular risk and mortality in hemodialysis patients. Kidney Int.

[3] Stenvinkel P, Heimburger O, Paultre F, Diczfalusy U, Wang T, Berglund L, Jogestrand T (1999). Strong association between malnutrition, inflammation, and atherosclerosis in chronic renal failure. Kidney Int.

[4] Qureshi AR, Alvestrand A, Divino-Filho JC, Gutierrez A, Heimburger O, Lindholm B, Bergstrom J (2002). Inflammation, malnutrition, and cardiac disease as predictors of mortality in hemodialysis patients. J Am Soc Nephrol.

[5] Rosin DL, Okusa MD (2011). Dangers within: DAMP responses to damage and cell death in kidney disease. J Am Soc Nephrol.

[6] Kuwabara T, Mori K, Mukoyama M, Kasahara M, Yokoi H, Saito Y, Ogawa Y, Imamaki H, Kawanishi T, Ishii A (2012). Exacerbation of diabetic nephropathy by hyperlipidaemia is mediated by toll-like receptor 4 in mice. Diabetologia.

[7] Lin M, Yiu WH, Li RX, Wu HJ, Wong DW, Chan LY, Leung JC, Lai KN, Tang SC (2013). The TLR4 antagonist CRX-526 protects against advanced diabetic nephropathy. Kidney Int.

[8] Lin M, Yiu WH, Wu HJ, Chan LY, Leung JC, Au WS, Chan KW, Lai KN, Tang SC (2012). Toll-like receptor 4 promotes tubular inflammation in diabetic nephropathy. J Am Soc Nephrol.

[9] Summers SA, van der Veen BS, O'Sullivan KM, Gan PY, Ooi JD, Heeringa P, Satchell SC, Mathieson PW, Saleem MA, Visvanathan K (2010). Intrinsic renal cell and leukocyte-derived TLR4 aggravate experimental anti-MPO glomerulonephritis. Kidney Int.

[10] Giorgini A, Brown HJ, Sacks SH, Robson MG (2010). Toll-like receptor 4 stimulation triggers crescentic glomerulonephritis by multiple mechanisms including a direct effect on renal cells. Am J Pathol.

[11] Odink K, Cerletti N, Bruggen J, Clerc RG, Tarcsay L, Zwadlo G, Gerhards G, Schlegel R, Sorg C (1987). Two calcium-binding proteins in infiltrate macrophages of rheumatoid arthritis. Nature.

[12] Korndorfer IP, Brueckner F, Skerra A (2007). The crystal structure of the human (S100A8/S100A9)2 heterotetramer, calprotectin, illustrates how conformational changes of interacting alpha-helices can determine specific association of two EF-hand proteins. J Mol Biol.

[13] Vogl T, Tenbrock K, Ludwig S, Leukert N, Ehrhardt C, van Zoelen MA, Nacken W, Foell D, van der Poll T, Sorg C (2007). Mrp8 and Mrp14 are endogenous activators of toll-like receptor 4, promoting lethal, endotoxin-induced shock. Nat Med.

[14] Croce K, Gao H, Wang Y, Mooroka T, Sakuma M, Shi C, Sukhova GK, Packard RR, Hogg N, Libby P (2009). Myeloid-related protein-8/14 is critical for the biological response to vascular injury. Circulation.

[15] Loser K, Vogl T, Voskort M, Lueken A, Kupas V, Nacken W, Klenner L, Kuhn A, Foell D, Sorokin L (2010). The toll-like receptor 4 ligands Mrp8 and Mrp14 are crucial in the development of autoreactive CD8+ T cells. Nat Med.

[16] Kuwabara T, Mori K, Kasahara M, Yokoi H, Imamaki H, Ishii A, Koga K, Sugawara A, Yasuno S, Ueshima K (2014). Predictive significance of kidney myeloid-related protein 8 expression in patients with obesity- or type 2 diabetes-associated kidney diseases. PLoS One.

[17] Pepper RJ, Hamour S, Chavele KM, Todd SK, Rasmussen N, Flint S, Lyons PA, Smith KG, Pusey CD, Cook HT (2013). Leukocyte and serum S100A8/S100A9 expression reflects disease activity in ANCA-associated vasculitis and glomerulonephritis. Kidney Int.

[18] Kawasaki Y, Hosoya M, Takahashi A, Isome M, Tanji M, Suzuki H (2005). Myeloid-related protein 8 expression on macrophages is a useful prognostic marker for renal dysfunction in children with MPGN type 1. Am J Kidney Dis.

[19] Heller F, Frischmann S, Grunbaum M, Zidek W, Westhoff TH (2011). Urinary calprotectin and the distinction between prerenal and intrinsic acute kidney injury. Clin J Am Soc Nephrol.

[20] Seibert FS, Pagonas N, Arndt R, Heller F, Dragun D, Persson P, Schmidt-Ott K, Zidek W, Westhoff TH (2013). Calprotectin and neutrophil gelatinase-associated lipocalin in the differentiation of pre-renal and intrinsic acute kidney injury. Acta Physiol (Oxf).

[21] Poon PY, Szeto CC, Kwan BC, Chow KM, Leung CB, Li PK (2012). Relationship between myeloid-related protein 8/14 and survival of Chinese peritoneal dialysis patients. Kidney blood Press Res.

[22] Morinaga J, Kakuma T, Fukami H, Hayata M, Uchimura K, Mizumoto T, Kakizoe Y, Miyoshi T, Shiraishi N, Adachi M, et al. Circulating angiopoietin-like protein 2 levels and mortality risk in patients receiving maintenance hemodialysis: a prospective cohort study. Nephrol Dial Transplant. 2019; *in press*. 10.1093/ndt/gfz236 31840173

[23] Arima H, Kubo M, Yonemoto K, Doi Y, Ninomiya T, Tanizaki Y, Hata J, Matsumura K, Iida M, Kiyohara Y (2008). High-sensitivity C-reactive protein and coronary heart disease in a general population of Japanese: the Hisayama study. Arterioscler Thromb Vasc Biol.

[24] Sekimoto R, Kishida K, Nakatsuji H, Nakagawa T, Funahashi T, Shimomura I (2012). High circulating levels of S100A8/A9 complex (calprotectin) in male Japanese with abdominal adiposity and dysregulated expression of S100A8 and S100A9 in adipose tissues of obese mice. Biochem Biophys Res Commun.

[25] Fukagawa M, Yokoyama K, Koiwa F, Taniguchi M, Shoji T, Kazama JJ, Komaba H, Ando R, Kakuta T, Fujii H (2013). Clinical practice guideline for the management of chronic kidney disease-mineral and bone disorder. Ther Apher Dial.

[26] Brun JG, Jonsson R, Haga HJ (1994). Measurement of plasma calprotectin as an indicator of arthritis and disease activity in patients with inflammatory rheumatic diseases. J Rheumatol.

[27] De Rycke L, Baeten D, Foell D, Kruithof E, Veys EM, Roth J, De Keyser F (2005). Differential expression and response to anti-TNFalpha treatment of infiltrating versus resident tissue macrophage subsets in autoimmune arthritis. J Pathol.

[28] Lugering N, Stoll R, Kucharzik T, Schmid KW, Rohlmann G, Burmeister G, Sorg C, Domschke W (1995). Immunohistochemical distribution and serum levels of the Ca (2+)-binding proteins MRP8, MRP14 and their heterodimeric form MRP8/14 in Crohn's disease. Digestion.

[29] Tibble JA, Bjarnason I (2001). Non-invasive investigation of inflammatory bowel disease. World J Gastroenterol.

[30] Schonthaler HB, Guinea-Viniegra J, Wculek SK, Ruppen I, Ximenez-Embun P, Guio-Carrion A, Navarro R, Hogg N, Ashman K, Wagner EF (2013). S100A8-S100A9 protein complex mediates psoriasis by regulating the expression of complement factor C3. Immunity.

[31] Roth J, Teigelkamp S, Wilke M, Grun L, Tummler B, Sorg C (1992). Complex pattern of the myelo-monocytic differentiation antigens MRP8 and MRP14 during chronic airway inflammation. Immunobiology.

[32] Mezzano D, Pais EO, Aranda E, Panes O, Downey P, Ortiz M, Tagle R, Gonzalez F, Quiroga T, Caceres MS (2001). Inflammation, not hyperhomocysteinemia, is related to oxidative stress and hemostatic and endothelial dysfunction in uremia. Kidney Int.

[33] Long J, Vela Parada X, Kalim S (2018). Protein Carbamylation in chronic kidney disease and Dialysis. Adv Clin Chem.

[34] Bohler J, Donauer J, Birmelin M, Schollmeyer PJ, Horl WH (1993). Mediators of complement-independent granulocyte activation during haemodialysis: role of calcium, prostaglandins and leukotrienes. Nephrol Dial Transplant.

[35] Yeun JY, Levine RA, Mantadilok V, Kaysen GA (2000). C-reactive protein predicts all-cause and cardiovascular mortality in hemodialysis patients. Am J Kidney Dis.

[36] Kaptoge S, Di Angelantonio E, Lowe G, Pepys MB, Thompson SG, Collins R, Danesh J (2010). C-reactive protein concentration and risk of coronary heart disease, stroke, and mortality: an individual participant meta-analysis. Lancet.

[37] Kuro OM (2013). A phosphate-centric paradigm for pathophysiology and therapy of chronic kidney disease. Kidney Int Suppl.

[38] Smith ER, Ford ML, Tomlinson LA, Rajkumar C, McMahon LP, Holt SG (2012). Phosphorylated fetuin-A-containing calciprotein particles are associated with aortic stiffness and a procalcific milieu in patients with pre-dialysis CKD. Nephrol Dial Transplant.

[39] Hamano T, Matsui I, Mikami S, Tomida K, Fujii N, Imai E, Rakugi H, Isaka Y (2010). Fetuin-mineral complex reflects extraosseous calcification stress in CKD. J Am Soc Nephrol.

[40] Kuro-o M (2013). Klotho, phosphate and FGF-23 in ageing and disturbed mineral metabolism. Nat Rev Nephrol.

[41] Koppert S, Buscher A, Babler A, Ghallab A, Buhl EM, Latz E, Hengstler JG, Smith ER, Jahnen-Dechent W (2018). Cellular clearance and biological activity of Calciprotein particles depend on their maturation state and Crystallinity. Front Immunol.

[42] Kuwabara T, Mori K, Mukoyama M, Kasahara M, Yokoi H, Nakao K (2014). Macrophage-mediated glucolipotoxicity via myeloid-related protein 8/toll-like receptor 4 signaling in diabetic nephropathy. Clin Exp Nephrol.

[43] Kraakman MJ, Lee MK, Al-Sharea A, Dragoljevic D, Barrett TJ, Montenont E, Basu D, Heywood S, Kammoun HL, Flynn M (2017). Neutrophil-derived S100 calcium-binding proteins A8/A9 promote reticulated thrombocytosis and atherogenesis in diabetes. J Clin Invest.

[44] Jimenez ZN, Silva BC, Reis LD, Castro MC, Ramos CD, Costa-Hong V, Bortolotto LA, Consolim-Colombo F, Dominguez WV, Oliveira IB (2016). High dialysate calcium concentration may cause more sympathetic stimulus during hemodialysis. Kidney Blood Press Res.

[45] Panichi V, Maggiore U, Taccola D, Migliori M, Rizza GM, Consani C, Bertini A, Sposini S, Perez-Garcia R, Rindi P (2004). Interleukin-6 is a stronger predictor of total and cardiovascular mortality than C-reactive protein in haemodialysis patients. Nephrol Dial Transplant.

[46] Nishikawa Y, Kajiura Y, Lew JH, Kido JI, Nagata T, Naruishi K (2017). Calprotectin induces IL-6 and MCP-1 production via toll-like receptor 4 signaling in human gingival fibroblasts. J Cell Physiol.

[47] Malickova K, Brodska H, Lachmanova J, Dusilova Sulkova S, Janatkova I, Mareckova H, Tesar V, Zima T (2010). Plasma calprotectin in chronically dialyzed end-stage renal disease patients. Inflamm Res.

