# Supplementary material for: The predictive role of serum calprotectin on mortality in hemodialysis patients with high phosphoremia
Source: BMC Nephrol. 2020 May 4;21:158. doi: 10.1186/s12882-020-01812-x (PMC7197146; doi:10.1186/s12882-020-01812-x)
Supplement: Supplementary file 1 — Additional file 1: S1 Table. Statistical analyses. S2 Table. Causes of death in this cohort. S3 Table. Multivariate adjusted hazard ratios in all-cause mortality according to CPT, hs-CRP and WBC tertiles in strata by clinical parameters reflecting bone-marrow function and nutritional status. Figure S1. Flow chart of participants in this study. Figure S2. 2D-scatter plot between clinical parameters and CPT at baseline. Figure S3. 2D-scatter plot between ABI and CPT at baseline for total cohort and in stratum of IP. [file 12882_2020_1812_MOESM1_ESM.doc]

**SUPPLEMENTAL MATERIAL**

**SUPPLEMENTAL TABLES**

**S1 Table.**

**Statistical analyses**

| **Results** | **Statistical methods** |
| --- | --- |
| Table 1 | - |
| Table 2 | Wilcoxon rank sum test |
| Table 3 | Spearman's correlation coefficient |
| Fig 1 | Spearman's correlation coefficient |
| Fig 2 | Kaplan-Meier survival analysis and multivariate Cox regression analysis |
| Fig 3 | Kaplan-Meier survival analysis and multivariate Cox regression analysis |
| Fig 4 | Kaplan-Meier survival analysis and multivariate Cox regression analysis |
| S1 Table | - |
| S2 Table | - |
| S3 Table | multivariate Cox regression analysis |
| S4 Table | multivariate Cox regression analysis |
| Figure S1 | - |
| Figure S2 | Spearman's correlation coefficient |
| Figure S3 | Spearman's correlation coefficient |

**S2 Table.**

Causes of death in this cohort

| Causes of death | n (%) |
| --- | --- |
| Cardiovascular disease | 27 (22.9) |
| Ischemic heart disease | 0 (0) |
| Cerebrovascular disease | 9 (7.6) |
| Other cardiovascular disease (CHF, AS, arrhythmia, sudden death) | 18 (15.3) |
| Infectious disease | 40 (33.9) |
| Cancer | 19 (16.1) |
| Other | 12 (10.2) |
| Unknown | 20 (16.9) |
| Total | 118 (100) |

CHF, congestive heart failure; AS, aortic stenosis

**S3 Table.**

**Multivariate adjusted hazard ratios in all-cause mortality according to CPT, hs-CRP and WBC tertiles in strata by clinical parameters reflecting bone-marrow function and nutritional status.**

In multivariate analysis, the model was adjusted for relevant covariates: age, sex, diabetes, rheumatoid arthritis, active cancer, history of coronary artery disease, albumin, Cr, clinics and inflammatory parameters. CPT, calprotectin; hs-CRP, high sensitivity C-reactive protein; WBC, white blood cells; Cr, Creatinine; Hb, hemoglobin; Plt, platelets; TG, triglyceride; IP, inorganic phosphorus. Tertiles of CPT, <4635.04, 4635.04–7915.68, >7915.68 ng/ml; hs-CRP, <0.427, 0.427–1.173, >1.173 mg/l; WBC, <4756.67, 4756.67–6156.67, >6156.67 /μl. Regarding the continuous variables, patients were stratified either by the median or by cut-off points into two groups. The median WBC is 5405 /μl, the median Plt is 15.9 104 /μl, the median Albumin is 3.8 g/dl. Stratification by Hb [1], BMI [2], Albumin [3], TG [4] and IP [5] employed the cut-off value in accordance with guidelines edited by Japanese Society of Dialysis Therapy. The cut-off value of Hb is 10.0 g/dl, BMI is 25 kg/m2, Albumin is 3.8 g/dl, TG is 150 mg/dl and IP is 6.0 mg/dl

**SUPPLEMENTAL FIGURES AND LEGENDS**

**Figure S1**

**Flow chart of participants in this study.**


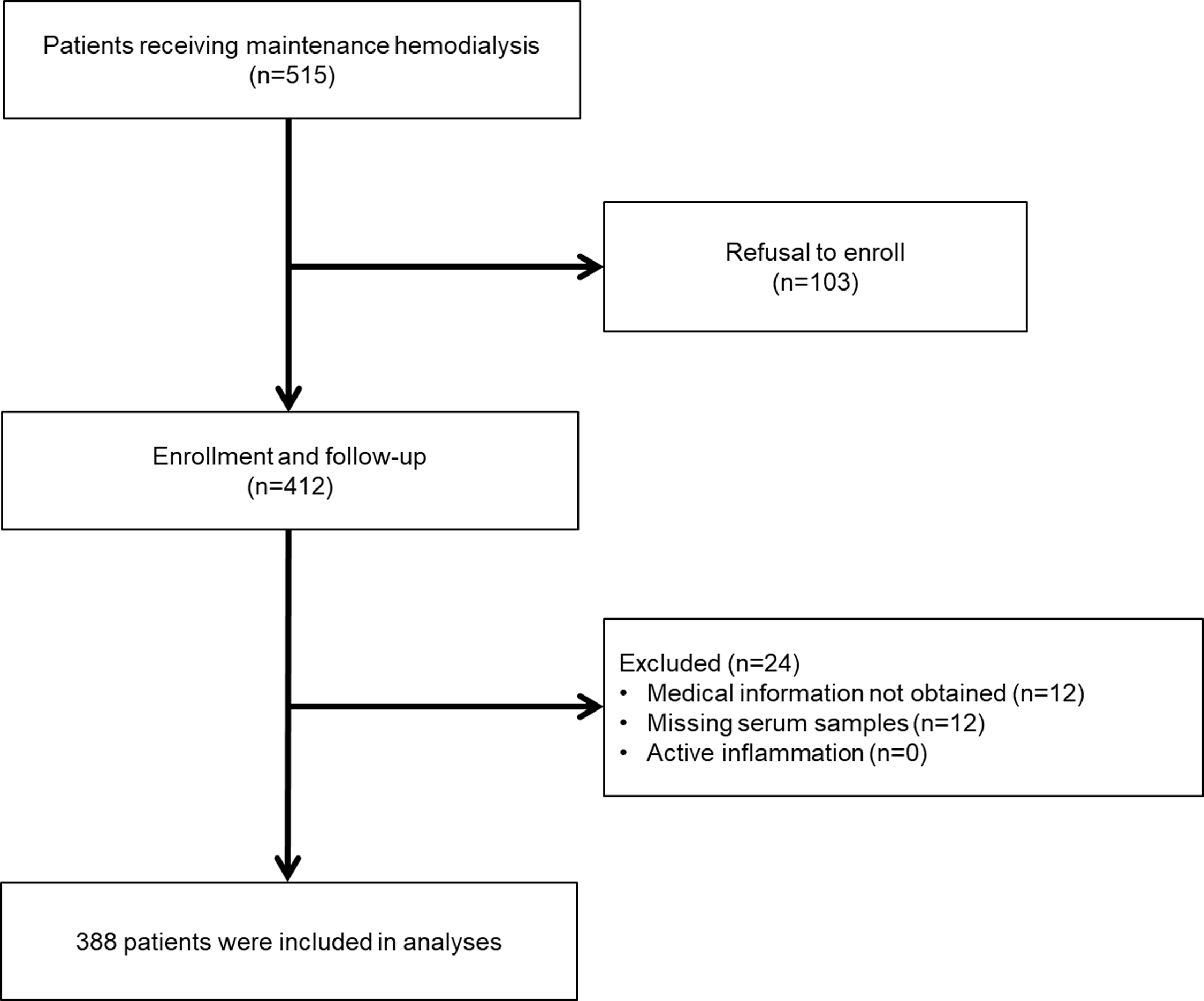


**Figure S2**

**2D-scatter plot between clinical parameters and CPT at baseline.**

Correlation between CPT and TG (A), IP (B) and BMI (C). CPT, calprotectin; TG, triglyceride; IP, inorganic phosphorus; BMI, body mass index.


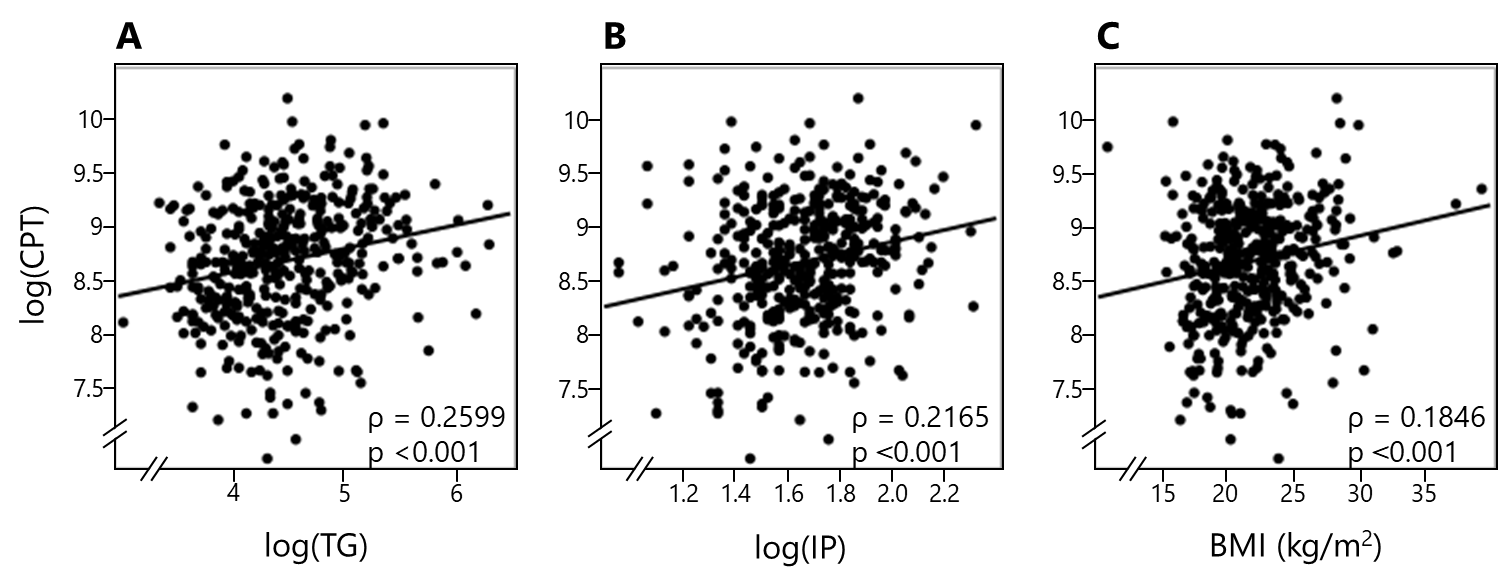


**Figure S3**

**2D-scatter plot between ABI and CPT at baseline for total cohort and in stratum of IP.**

Correlation between ABI and CPT. Total cohort (A), stratum of IP<6.0mg/dl (B), stratum of IP≥6.0mg/dl (C). ABI, Ankle-Brachial-Index ; IP, inorganic phosphorus; CPT, calprotectin


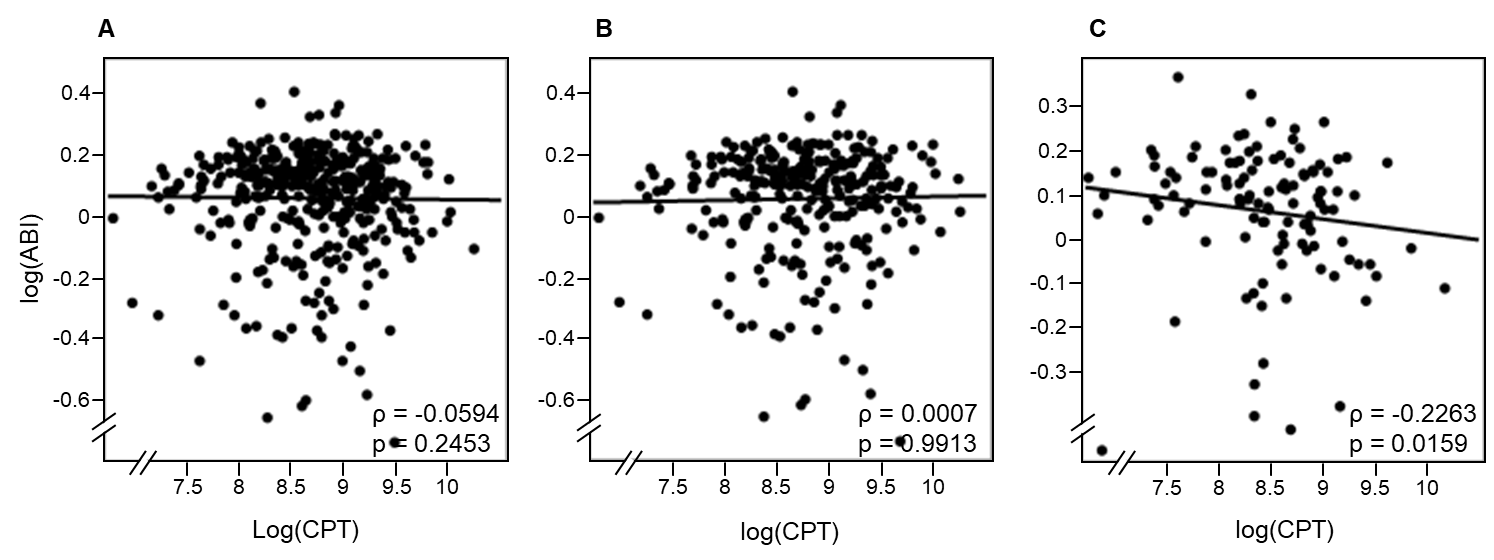


**References**

1. Shoji T, Niihata K, Fukuma S, Fukuhara S, Akizawa T, Inaba M: **Both low and high serum ferritin levels predict mortality risk in hemodialysis patients without inflammation**. *Clin Exp Nephrol* 2017, **21**(4):685-693.

2. **Appropriate body-mass index for Asian populations and its implications for policy and intervention strategies**. *Lancet* 2004, **363**(9403):157-163.

3. Kalantar-Zadeh K, Kilpatrick RD, Kuwae N, McAllister CJ, Alcorn H, Jr., Kopple JD, Greenland S: **Revisiting mortality predictability of serum albumin in the dialysis population: time dependency, longitudinal changes and population-attributable fraction**. *Nephrol Dial Transplant* 2005, **20**(9):1880-1888.

4. Hirakata H, Nitta K, Inaba M, Shoji T, Fujii H, Kobayashi S, Tabei K, Joki N, Hase H, Nishimura M *et al*: **Japanese Society for Dialysis Therapy guidelines for management of cardiovascular diseases in patients on chronic hemodialysis**. *Ther Apher Dial* 2012, **16**(5):387-435.

5. Fukagawa M, Yokoyama K, Koiwa F, Taniguchi M, Shoji T, Kazama JJ, Komaba H, Ando R, Kakuta T, Fujii H *et al*: **Clinical practice guideline for the management of chronic kidney disease-mineral and bone disorder**. *Ther Apher Dial* 2013, **17**(3):247-288.
